# Logistic Mixed Models to Investigate Implicit and Explicit Belief Tracking

**DOI:** 10.3389/fpsyg.2016.01681

**Published:** 2016-11-02

**Authors:** Martin Lages, Anne Scheel

**Affiliations:** ^1^School of Psychology, University of GlasgowGlasgow, UK; ^2^Department of Psychology, Ludwig-Maximilians-Universität MünchenMünich, Germany

**Keywords:** Theory of Mind (ToM), logistic regression, mixed models, eye gaze, decision making

## Abstract

We investigated the proposition of a two-systems Theory of Mind in adults’ belief tracking. A sample of *N* = 45 participants predicted the choice of one of two opponent players after observing several rounds in an animated card game. Three matches of this card game were played and initial gaze direction on target and subsequent choice predictions were recorded for each belief task and participant. We conducted logistic regressions with mixed effects on the binary data and developed Bayesian logistic mixed models to infer implicit and explicit mentalizing in true belief and false belief tasks. Although logistic regressions with mixed effects predicted the data well a Bayesian logistic mixed model with latent task- and subject-specific parameters gave a better account of the data. As expected explicit choice predictions suggested a clear understanding of true and false beliefs (TB/FB). Surprisingly, however, model parameters for initial gaze direction also indicated belief tracking. We discuss why task-specific parameters for initial gaze directions are different from choice predictions yet reflect second-order perspective taking.

## Introduction

In order to understand the thoughts and beliefs of others we have to make inferences from their behavior. Consider observing a person who walks down the street, suddenly stops, turns around and walks into the opposite direction. We may infer that this person just remembered something important leading to a change of plans. This kind of reasoning about another person’s mental state, is called ‘mentalizing’ or ‘Theory of Mind’ (ToM; Premack and Woodruff, 1978). Having a ToM in this context means appreciating that others follow their own goals and beliefs and may change their mind or mental states. These states are not directly observable but may be inferred from behavioral (Lee and Homer, 1999) and neural representations (Schaafsma et al., 2015).

In this paper we first describe the idea of implicit and explicit belief tracking before we derive critical hypotheses. We then establish a novel method for investigating belief tracking in a game between two players. We conducted a first experiment on belief tracking in adults and analyzed the binary data using different types of logistic mixed models.

A general problem for testing hypotheses about belief tracking is that the probability for a belief in a specific task and situation needs to be inferred from the performance of participants whose understanding of the task and scenario may vary. Repetition of the same belief task should be avoided because this may lead to beliefs being recalled by participants. Other typical issues in studies on ToM and belief tracking are that missing data are relatively common and that the sample size tends to be small.

Non-parametric tests often lack power, have difficulties to accommodate the full experimental design, and do not acknowledge variability across participants. A better approach is to model binary data in a logistic regression with mixed effects. These models can determine task-specific fixed and subject-specific random effects in most experimental designs. Bayesian logistic mixed models, however, can predict individual behavior by using weighted combinations of latent task-specific and subject-specific parameters. Estimating latent parameters in Bayesian logistic mixed models provides a more flexible approach than logistic regressions because these models can estimate missing values and accommodate individual distortions of belief probabilities.

### Mentalizing and Belief Tracking

Mentalizing is an important social cognitive ability that we frequently use. It helps us to compete, co-operate, and communicate with others. Mental states include different motivational, emotional, and cognitive experiences such as goals, desires, preferences, and beliefs. Beliefs are so-called epistemic mental states because they are a representation of a state of reality that can be true or false.

Tracking mental states such as goals and preferences appears to be a relatively simple task that infants develop in their first months (Gergely et al., 1995; Repacholi and Gopnik, 1997; Woodward, 1998; Sommerville et al., 2005). Tracking true beliefs (TBs) is relatively easy because the person’s representation of reality is congruent with reality and with their own perspective. Tracking false beliefs (FBs) can be more challenging because it requires monitoring reality (as it is subjectively perceived) and another person’s incorrect representation of reality. This is why false-belief tasks have long been considered as the ‘litmus test’ for a fully developed ToM (Dennett, 1979; Wimmer and Perner, 1983; Lee and Homer, 1999). Over two decades, numerous studies have established that 4- to 5-year-olds succeed in explicit false-belief tests whereas 3-year-olds consistently fail (Wellmann et al., 2001).

In sharp contrast to this, more recent studies using violation-of-expectation (VoE) paradigms have suggested that 13- to 15-month-olds may already have an implicit understanding of FB (Onishi and Baillargeon, 2005; Surian et al., 2007; Song et al., 2008; Träuble et al., 2010). With more direct measures of anticipation, the same pattern of results has been found in children up to 2 years before they first pass explicit false-belief tasks (Clements and Perner, 1994; Southgate et al., 2007).

### Two-Systems Theory

This 2- to 3-year gap between the first emergence of implicit tracking of beliefs and passing the explicit Sally–Anne test cannot be explained by language development only (for an overview see Lee and Homer, 1999).

It also speaks against traditional views of a continuous transition from a premature ToM system covering simple mental states to a mature system covering beliefs (Song et al., 2008; Carey, 2009; Sodian, 2011). Apperly and Butterfill (2009) offered a different and more integrative account. They suggested two ToM systems that are not mutually exclusive, but form two stages: an earlier-developing, *fast and efficient* but inflexible system that rapidly tracks belief-like states and a later-developing slow and complex but *flexible* system that can track various beliefs.

The fast and efficient system may be innate and shared with intelligent and social animals (e.g., Hare et al., 2006; Clayton and Emery, 2007). It allows tracking various mental states (including beliefs) spontaneously and effortlessly, but its efficiency comes at the cost of certain ‘signature limits’ (Apperly and Butterfill, 2009, p. 960). The flexible system is thought to emerge later in ontogeny and then to co-exist with the fast and efficient system. It covers almost any kind of mentalizing in different situations, but its flexibility requires additional cognitive resources. Typical human adults should possess both systems but may only use the fast and efficient system depending on factors such as task complexity, time pressure, cognitive load, and motivation.

According to this theory, infants have a system in place that can roughly compute others’ belief-like states in a number of everyday situations, long before they are able to explicitly reason about mental states. Adults on the other hand should be able to access the flexible system depending on task requirements in a given situation. This claim may be investigated by testing whether implicit belief-tracking abilities in adults have the same signature limits as infants. Here, we directly compare implicit belief-tracking followed by explicit mentalizing in adults only.

### Fast and Efficient Belief-Tracking

In order to define these signature limits, Apperly and Butterfill (2009) suggested three possible mechanisms of a fast and efficient system: ‘automatisation,’ behavioral associations, and so-called ‘registrations.’ Please note that the authors do not claim that this list is exhaustive. Although automatisation and behavioral associations cannot be ruled out, neither of these mechanisms is sufficient to explain the current body of empirical findings.

Apperly and Butterfill (2009) advocate registrations as the most plausible explanation for fast and efficient belief tracking. Registrations are thought to be a proxy for beliefs: they link an agent with an object and its properties (such as its location). Registrations are stable over time: for instance when an agent encountered an object in a certain location, this registration stays valid until the agent encounters the object in a new location. In the Sally–Anne test for example (Wimmer and Perner, 1983; Baron-Cohen et al., 1985), the observing participant would be able to track Sally’s FB by proxy of a registration: the link between Sally, her toy, and its first location would stay valid after Sally leaves the scene and until Sally realizes that her toy is no longer at its location.

However, Apperly and Butterfill (2009) do not claim that registrations provide an explanation for every kind of belief reasoning. In order to be more efficient than sophisticated mentalizing, they define the following limitations:

Registrations must be relations to objects and properties, not to propositions; and registrations must have their effects on action by setting parameters for action independently of each other and independent of any psychological states... Accordingly, registrations would support Level 1 perspective taking... but not Level 2 perspective taking. (p. 963)

According to the above quote a fast and efficient system that relies on registrations would have two main signature limits. It would not cover Level 2 perspective taking, i.e., understanding that an object can have a different identity or purpose for someone else (e.g., Gopnik and Astington, 1988), and it would not allow to track beliefs about mental states.

The first of these claims has been investigated and repeatedly confirmed. Infants and great apes seem to be capable of Level 1 perspective taking (Hare et al., 2000, 2001, 2006; Luo and Baillargeon, 2007; Sodian et al., 2007) but fail at Level 2 perspective taking (Call and Tomasello, 2005). In implicit measures such as eye gaze, adults and older children spontaneously track Level 1 but not Level 2 perspectives (Ferguson and Breheny, 2012; Surtees et al., 2012; Low and Watts, 2013). The second claim – that a fast and efficient system cannot track beliefs about mental states – has found less attention. This is surprising because a number of everyday social interactions require this type of mentalizing.

Imagine the following situation: a friend surprises you with a box of chocolates. Unfortunately, the chocolates are peppermint-flavored and you strongly dislike the taste of peppermint in chocolates. If you are sure that your friend does not know about your dislike, you may be grateful and feel touched, even though you do not like the chocolates. Conversely, if you are certain that your friend knows about your dislike, you may feel teased or even get upset.

This is an example of a social judgment that is based on implicit mentalizing: the donor of a gift is not only judged by their behavior but also by their beliefs and intentions. According to Apperly and Butterfill (2009), this kind of mentalizing would not be covered by a fast and efficient system based on registrations. As a consequence infants and great apes but also adults should be incapable of implicit belief tracking.

This last prediction is contradicted by research suggesting that even infants can make social judgments (Behne et al., 2005; Hamlin et al., 2007, 2013). In a study with 10-month-olds (Hamlin et al., 2013), the infants were asked to choose between two elephant puppets after they had observed the first elephant helping a lion puppet to achieve its goal and the second elephant being not helpful (although both elephants performed the same motor action). Critically, the elephant did or did not know about the lion’s goal. Infants preferred the helpful elephant to the unhelpful one *only* when the elephant knew about the lion’s goal. To judge the elephants as helpful or unhelpful, infants had to infer goals or intentions from the behavior of the elephants *while* taking into account the elephants’ belief about the lion’s goal. In other words, they had to track beliefs about mental states – something a fast and efficient ToM system that relies on registrations would not allow them to do.

Because these findings are at odds with one specific aspect of an otherwise elegant theory, it seems worthwhile to investigate this issue further. The main claim of the two-system theory is the co-existence of two ToM systems, one of which is fast and efficient whereas the second one is slow but flexible. The fast and efficient system comes at the cost of certain signature limits, and has been observed in infants and some primates by recording initial eye gaze. The slow and explicit system on the other hand requires cognitive effort and should be present in 5–6 year-old children as well as adults when making explicit choice predictions.

If the two-systems ToM is a valid model, adults’ implicit belief tracking may parallel infants’ performance in the study by Hamlin et al. (2013). If this is the case, the signature limits of the fast and efficient system should be reconsidered.

On the other hand, if adults’ implicit belief tracking is similar to their explicit performance or different from the results by Hamlin et al. (2013) then the idea of a two-systems ToM may be called into question altogether.

### Belief Tracking in a Card Game

**FIGURE 1:**
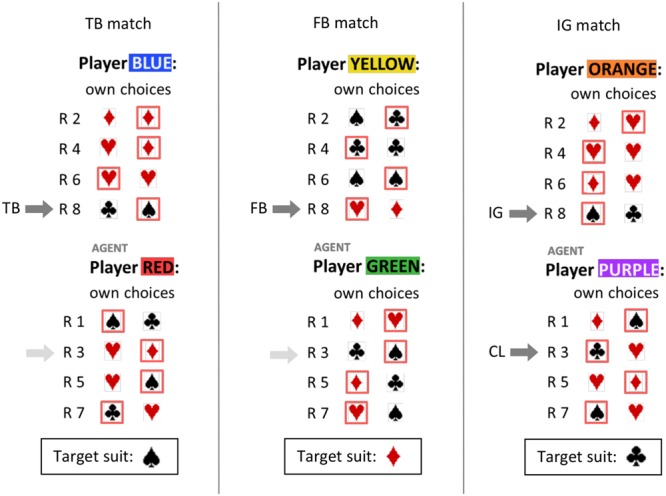
**Schematic overview of the two cards displayed in alternating rounds (R1–R8) in the True Belief (TB), False Belief (FB), and Ignorance (IG/CL) match.** Each choice is illustrated by a frame around the suit on the left- or right-hand side.

In the present study, we suggest a novel method to investigate adults’ implicit and explicit belief tracking abilities. For this purpose we devised a simple card game between two players (see **Box 1** and **Figure 1**). Three different matches were played to model situations in which one of the players (Player 2) either holds a TB or a FB about the goal of the other player (Player 1). In a third game Player 2 may remain ignorant (IG) about the goal of Player 1 but the IG match also included a control condition (CL).

**Box 1. Rules of the card game as explained to participants.**

Players, Cards, and Objective

• Two players;
• Standard 52 card pack (without Jokers);
• A game consists of two ‘sets’ with eight rounds each;
• In each set, one of the players is the ‘Agent’: Player 1 in Set 1, Player 2 in Set 2. The Agent first chooses one of the four suits as the ‘target suit.’ The choice is recorded but *not revealed to the other player*. It cannot be changed during the set;
• The goal for both players is to collect as many cards of the target suit (target cards) as possible.

Play and Scoring

• In each round, two cards are drawn from a shuﬄed deck and placed face-up on a table.
• The players take turns in picking the first card: The Agent picks first in Round 1, the other player picks first in Round 2, and so on. In each round, the remaining card is automatically assigned to the other player. This means, each player collects one card in each round, but can only actively choose a card in every other round.
• The collected cards are recorded. The cards are put back into the deck and shuﬄed, so that every card is equally likely to be drawn from the deck in every round.
• At the end of a set the target suit is revealed. Each player receives one point for each target card they collected.
• At the end of the game the total scores from both sets are compared. The player with more points wins the game but a draw is also possible.

In Set 1 the Agent (Player 1) always knows the target suit whereas the other player (Player 2) has to infer the target suit from the Agent’s active card choices.

By asking participants to predict which of two cards a player is going to choose, it is possible to test how accurately they track different belief states. Unknown to the participants their initial eye gaze was recorded by a built-in camera on top of the display when a target and non-target card appeared on the left and right-hand side of the screen.

This implementation has several advantages compared to classic perspective-taking tasks (e.g., Low and Watts, 2013). A card game is an interactive and engaging situation most adults are familiar with. Most card games require some form of perspective taking and deceptive strategies are also relatively common. This helps to avoid certain demand characteristics in an experimental setup where adults may expect the agent to be “omniscient.” Observing a card game between two players clearly suggests that the beliefs of the two players involved may differ from each other. Because the experimenter is in full control of the displayed cards and the players’ choices, the game is a versatile tool to measure flexibility in thinking and to convey different belief states of players (Berg, 1948; Grant and Berg, 1948). For the purpose of this study, we tried to establish three scenarios suggesting a state of “TB,” “FB,” and “ignorance” in the second player, but other scenarios are possible.

Importantly, participants did not play the game themselves, but watched two players who chose between two “randomly drawn” cards in successive rounds. For each player the objective of the game was to collect as many cards as possible from the target suit (see **Box 1** for detailed rules). At the beginning of a match, Player 1 decided which suit was the target suit and should be collected to gain points, whereas Player 2 was not informed about this choice. Player 1 and the participant therefore held superior knowledge about the goal of the game. In order to gain as many points as possible, Player 2 had to infer the goal from the choices Player 1 made. Depending on the cards and the choices of Player 1 the belief state of Player 2 may change over time from ignorance to TB or FB.

A FB can occur if Player 1 is deceptive and deliberately leaves a target card to Player 2 at the beginning of a match. This strategy can be more advantageous for Player 1 than immediately collecting a target card in the first round because it increases uncertainty about the target suit in Player 2. As a consequence Player 1 may passively collect target cards in subsequent rounds as long as Player 2 remains ignorant about the target suit.

However, FB in Player 2 may also occur in the IG match. If Player 1 is not deceptive but collects the same non-target suit then Player 2 may develop a FB about the target suit (see Discussion). The IG match also included a control condition (CL) in Round 3. In the CL task the participant had to predict the choice of Player 1 who had selected the target suit in Round 1. Since the participant was informed about the target suit the choice prediction in CL should reflect simple first-order perspective taking.

### Design

Participants watched three different matches of this card game. The matches were played so that in the last round of Set 1 (R8), Player 2 (BLUE) should hold a TB about Player 1’s (RED) target suit (Spades), Player 2 (YELLOW) should hold a FB about Player 1’s (GREEN) target suit (Hearts), and Player 2 (ORANGE) should be ignorant (IG) about Player 1’s (PURPLE) target suit (Clubs). Before they observed Player 2’s move in Round 8, participants were asked to predict which card Player 2 would choose. They also indicated on a five-point Likert scale how certain they were about their answer (0 = not at all certain, 4 = very certain). Before this question was displayed on screen and while participants were expecting to see one of the cards move toward Player 2 as in previous rounds, their eye gaze was secretly recorded by a built-in video camera in the computer monitor. Their initial eye gaze was later coded as on/off target in a binary scheme.

As a control (CL) the same procedure was applied to Round 3 of the IG match in order to monitor first-order perspective taking. First-order perspective taking occurred when Player 1 (PURPLE) chose between a target card and a non-target card. To keep the procedure comparable across matches, participants were asked to predict the move of Player 1 (RED, GREEN) in Round 3 of the TB and FB matches as well. In these rounds however, none of the cards were target cards and participants’ predictions were recorded but not analyzed.

The order of the three matches was varied across participants to account for possible sequence effects. Each participant was randomly assigned to a different sequence of tasks. Note that the CL task always occurred in Round 3 of the IG match.

### Experimental Hypotheses

Our first hypothesis concerned the question whether a single or two-systems ToM is used for initial eye gaze and subsequent choice predictions. If gaze directions and choice predictions correspond closely then a single ToM system may explain the data. If the two measures are sufficiently different then they may be governed by two distinct systems.

More specifically, if adults’ implicit belief tracking abilities are determined by a fast and efficient ToM system and if this system does not allow tracking of beliefs about mental states (Apperly and Butterfill, 2009), then initial eye gaze on target should be the same for TB, FB, and IG task but different from the CL task. On target predictions in the CL task reflect the system’s ability for first-order perspective taking (‘Registrations hypothesis’).

If the fast and efficient system can track beliefs about mental states, in line with the findings by Hamlin et al. (2013), then gaze responses should be above chance level in the TB (and CL task) and below chance level (off target) in the FB and IG task (‘Alternative hypothesis’). Finally, if there is no specific system for implicit mentalizing, gaze responses should either be at chance or above chance in all tasks reflecting an egocentric bias.

Adults’ explicit belief reasoning should be reasonably accurate. In order to make explicit choice predictions, participants were provided with all relevant information, had no time pressure, and received a monetary reward of €0.50 for every correct answer. Depending on the participants’ degree of certainty in each match, choice predictions on target should be clearly above chance (on target) in the TB and CL task, and clearly below chance (off target) in the FB and IG task. The prediction that the IG task also leads to below chance probabilities is a consequence of the similarities between the FB and IG match: as in the FB match Player 1 collected a non-target suit twice in earlier rounds (spades in **Figure 1**). It is therefore reasonable to assume that Player 2 did not remain ignorant but developed a FB about the target suit.

## Materials and Methods

### Participants

A sample of *N* = 45 adult participants took part. Participants were recruited from an online subject pool of the School of Psychology, University of Glasgow. Two subjects were replaced because they accidentally skipped at least one of the test rounds in the presentation. The final sample consisted of 33 females and 12 males with a mean age of 22.6 years (*SD* = 3.74, age range: 18–38 years). Participants were mainly undergraduate students (39 undergraduates, 3 postgraduates, 3 doctoral students) and came from 19 different countries. Subjects were invited to participate in individual sessions and received €3–€6 for their participation (depending on their performance, as detailed below).

### Procedure

Subjects were told that the study was about perspective taking and gave written consent to participate. To ensure that the eye gaze reflected implicit processes and was not biased by demand characteristics, subjects were not informed that their eye gaze would be recorded during the session. After the experiment, they were fully debriefed and made aware that they could withdraw their consent. Ethical approval for this procedure was obtained from the Glasgow University College of Science and Engineering ethics committee in line with the BPS Code of Ethics and Conduct for Human Research and Ethical Principles for Medical Research Involving Human Subjects of the WMA (Declaration of Helsinki). Participants received €3.00 for their participation and €0.50 for each correct prediction (out of six), so they could earn a total of up to €6.00.

The participant proceeded through each match by pressing the space bar on a keyboard. In the first part, the rules of the game and the participants’ task were explained in a demonstration match. In the second part, each participant watched three matches. Although a match consisted of two sets (so that each player has the advantage of being the agent in one set), participants only observed the first set of each match to keep the experiment short. They were told that they will have to predict the players’ choices in two rounds of each match and that they should try to anticipate the acting player’s choice of cards in each round. In fact, they were only asked to make a prediction in Round 3 and Round 8 (test rounds) of each match.

Two differently colored bars were displayed at the top and bottom of the screen symbolizing the two players. In each round, one of the colored bars was highlighted to indicate the acting player (agent). Then two cards were displayed side-by-side on the screen. In order to observe the active player’s choice, the participant pressed the space bar and one of the cards would move toward the player (up or down on the screen) to reveal the agent’s choice. Next, participants viewed a score panel displaying all previous rounds, the players’ choices up to the current round, and how many cards of each suit they had collected in total (actively or passively, see **Figure 1**). Note that only actively collected cards reveal an intention but that passively collected cards affect the total score.

In the test rounds, a blank screen appeared after participants pressed the space bar. After 2 s, they were asked to predict which of the two cards the active player was most likely to choose. Participants gave their choice prediction on a sheet of paper that showed the card table, the latest score, and a question about the certainty of their prediction. After filling out the response sheet they pressed the spacebar to continue the presentation. They watched the actual choice of Player 2 as immediate feedback on the correctness of their choice prediction.

To minimize influence from visual saliency cues on eye gaze, all cards in a match had the same number (TB match: 2s, FB match: 10s, IG match: 8s) and the two cards in Round 8 had the same color (Red or Black). The sequence of tasks and location of cards with correct predictions was counterbalanced.

After the presentations each participant’s personal data were collected, and they were debriefed and paid. All participants fully engaged in the card game and stated that the game and tasks were enjoyable and interesting.

### Materials

The experiment was run on a MacBook Pro and presented on a 27″ Apple Thunderbolt Display with a built-in high-resolution video camera. A chin rest at a viewing distance of 65 cm was used to ensure consistent quality of the video sequences. Participants’ faces and eye gaze were recorded using the built-in video camera of the display. The small green LED next to the camera was masked by black tape to conceal that the camera was in operation. ScreenFlow 4 was used to simultaneously record participants’ eye gaze and the presentation on screen.

### Video Analysis

**FIGURE 2:**
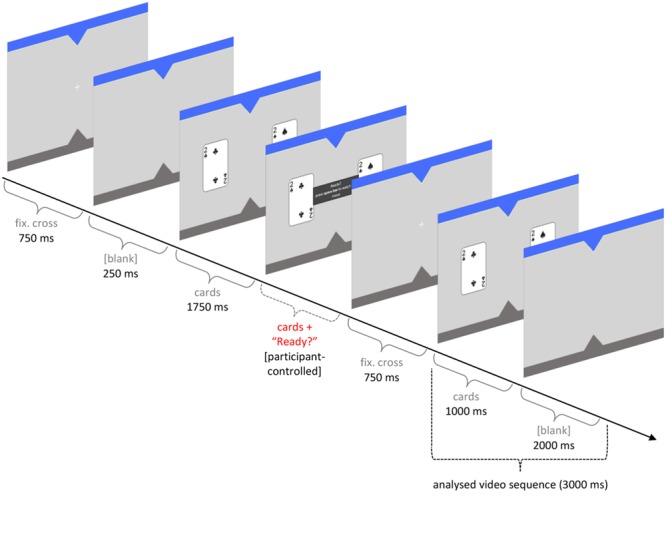
**Timeline of a test round indicating recording of initial eye gaze direction in a video sequence lasting 3 s**.

Each round started with a fixation cross at the center immediately followed by two cards on the left and right hand side. After 1750 ms, a panel appeared between the cards with the words ‘Ready? Press space bar to watch the move.’ The panel and the two cards remained visible and participants could view them as long as they wanted. Upon pressing the space bar, the cards disappeared and the fixation cross reappeared for 750 ms. The cards were then displayed again for 1000 ms. In a regular round, one of the cards then moved toward the active player to indicate the Agent’s choice. In a test round, the screen went blank for 2000 ms (see **Figure 2**). Then another panel appeared, asking participants to predict the choice of the Agent. Videos of the participant’s face were taken from the onset of the second time the cards were displayed to the end of the following blank screen (see **Figure 2**).

The video clips were analyzed by one of the authors (A.S.) and a researcher who was naïve about the hypotheses. File names were randomized so that both coders did not know to which task a given video belonged. Eye gaze was coded as the first saccade to the left or to the right and later re-coded to 1 = on target, 0 = off target. Inter-coder agreement was 96.8%. Disagreement in nine rounds was resolved by discussion. In 11 rounds, participants looked straight ahead during the video sequence and these recordings were treated as missing values (NA).

## Results

We first report results from conventional statistics and two logistic regressions models with mixed effects, followed by results from Bayesian logistic mixed models.

### Eye Gaze and Choice Predictions

**FIGURE 3:**
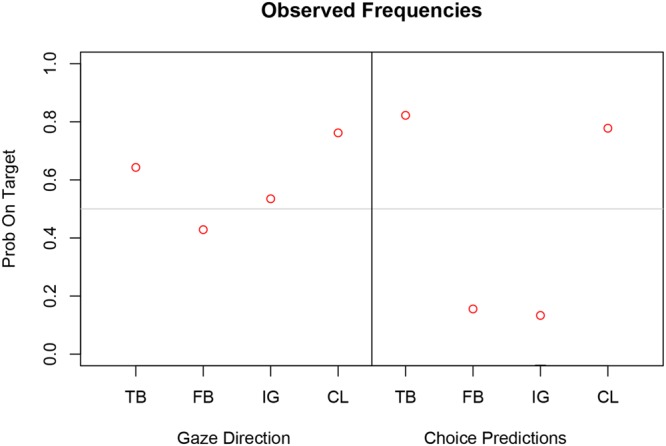
**Observed relative frequencies on target for each measure (**Left**: gaze direction; **Right**: choice prediction) and task (TB, true belief; FB, false belief; IG, ignorance; CL, control)**.

As expected, participants’ explicit prediction that the active player would choose the target card were above chance in the TB and CL task, and below chance in the FB and IG task (right panel in **Figure 3**). We performed non-parametric Pearson Chi-Square tests on the relative frequencies. The explicit choice predictions were all statistically significantly different from chance (*p* < 0.001).

For the relative frequencies of gaze direction on target, however, only CL was significantly different from chance [χ^2^(1) = 9.09, *p* < 0.003] whereas performance was around chance level (0.5) for TB, FB, and IG task (see **Table 1** and left panel in **Figure 3**). Thus the relative frequencies for gaze direction did not clearly discriminate between TB and FB and between Registration and Alternative hypothesis. This pattern of results speaks against an overall egocentric bias or overall chance performance, but it is unclear from the implicit measure whether perspective taking took place.

**Table 1:** Summary of logistic mixed models.

Model type	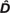	pD	DIC
**Bayesian logistic**			
Model 1	382.1	7.9	397.9
Model 2	362.1	16.9	395.9
Model 3	314.8	23.2	361.2
Model 4	280.4	55.6	391.6
**Logistic regression**			
lmer max	364.4	23	410.4
lmer pars	381.7	18	399.7

The 2 by 2 contingency tables for each task and measure were also computed. The results of McNemar tests on the contingency tables indicate statistically significant differences between implicit gaze directions and explicit choice predictions for the FB [χ^2^(1) = 5.88, *p* = 0.015] and Ignorance task [χ^2^(1) = 13.13, *p* = 0.0003] but not for TB [χ^2^(1) = 2.12, *p* = 0.146] and the Control task [χ^2^(1) = 0.1, *p* = 0.752]. These results suggest differences between tasks across measures but do not take into account the full experimental design of the study.

### Logistic Regression with Mixed Effects

To cover the full design of the study we conducted logistic regressions with mixed effects on implicit eye gaze and explicit choice prediction (R package lme4; Bates et al., 2014). A “maximal” model (lmer max) with fixed effects for task, measure and their interaction also had random slopes and intercepts for task and measure (Barr et al., 2013). This model gave a good fit in terms of the Akaike Information Criterion (AIC = 410.4, equivalent to the Deviance Information Criterion (DIC) for non-Bayesian models). A regression with the same fixed effects but random slopes for measure only (lmer pars) gave a more parsimonious fit in terms of AIC = DIC = 399.7 (Bates et al., 2015). Similar to the maximal model this regression analysis revealed statistically highly significant contrasts between CL task as the baseline and FB (*p* = 1.37e-09) and IG task (*p* = 1.96e-08) but no significant effect between CL and TB task (*p* = 0.679). In addition, there was a statistically significant effect of measure between eye gaze and choice prediction (*p* = 0.021). The contrasts between tasks were qualified by statistically significant interactions between eye gaze and choice prediction (measure) for FB vs CL (*p* = 0.039) and IG vs CL (*p* = 0.004) in line with the McNemar tests. Note that predictor variables (task, measure) were centered on the mean and dummy coded before they were entered into the regression analyses.

The logistic regression model captured fixed effects of task and measure as well as their interaction while assuming random slopes for measure. This mixed model produced the lowest AIC among regression models and the significant contrasts suggest differences in second-order perspective taking. Moreover, the significant main effect of measure and the significant interactions between tasks and measure indicate systematic differences between implicit and explicit belief tracking. The results are summarized in Appendix A.1.

Can we further reduce the predictive error (DIC) in a related Bayesian approach? For example, we specified a logistic regression model with random slopes and intercepts for each subject but it was not possible to establish a model with random slopes only. Bayesian logistic mixed models are more flexible and can estimate latent task- and subject-specific parameters under different constraints. Since these parameters are not directly observable they may not match the task-specific relative frequencies as shown in **Figure 3**. In addition, Bayesian models can estimate missing data rather than throwing away observations and Bayesian model selection offers a straightforward procedure to compare different model variants and to test the registrations and alternative hypothesis.

### Bayesian Logistic Mixed Models

Inspired by Rasch models and Item-Response Theory (IRT) we investigated Bayesian logistic mixed models with latent task-specific and subject-specific parameters. IRT assumes that latent variables such as item or task difficulty and subject ability determine observed performance. Here, we adopt this idea by applying a Bayesian logistic mixed model with weighted combinations of latent task-specific and subject-specific parameters to model initial eye gaze and choice predictions in our card game.

As for the logistic regressions we employ the logit function (inverse of the sigmoidal logistic function) on probability *p.*

$$\begin{array}{c} \mathrm{logit}(p)=ln\left(\frac{p}{1-p}\right) \end{array}$$

The model mixes latent variables 𝜃_i_ and α_j_ to predict relative frequencies p_ij_ of binary gaze direction and choice prediction (on target/off target). The mixing weight β_i_ determines in log odds how much an observer relies on task-specific probabilities on target as opposed to a default probability on target.

The latent parameter 𝜃_i_ reflects subject-specific inflection points for participant (*i* = 1–45) whereas α_j_ refers to task-specific parameters for gaze direction (*j* = 1–4) and choice prediction (*j* = 5–8) on target. In contrast to the logistic regressions we did not introduce ‘measure’ as a factor but simply establish probability estimates for all tasks and measures. Both parameters α_j_ and 𝜃_i_ are expressed in log odds and are combined linearly using a mixing weight β_i_. The mixing weight β_i_ is equivalent to the slope of a linear function in log-log space and models the relative contribution of subject-specific 𝜃_i_ and task-specific α_j_. This particular mixed model is based on the (ubiquitous) log-odds model (Zhang and Maloney, 2012) that itself is related to a large family of probability weighting functions (Tversky and Kahneman, 1974; Prelec, 1998; Gonzalez and Wu, 1999; Luce, 2000). Here, we use the log-odds model as a mixed model that combines task-specific parameters with subject-specific inflection points and mixing weights. For a recent application of the log-odds model to decision-making see Boos et al. (2016).

We established four possible model variants. The Bayesian Logistic Mixed Model 1 without subject-specific variability has task-specific parameters α_j_, mixing weight β, and inflection point 𝜃.

$$\begin{array}{c} \mathrm{logit}(k_{ij})\sim \;\beta \cdot \;\alpha_{j}+(1-\beta )\cdot \;\theta \end{array}$$

In Model 2, we introduced subject-specific inflection points 𝜃_i_.

$$\begin{array}{c} \mathrm{logit}(k_{ij})\sim \;\beta \cdot \;\alpha_{j}+(1-\beta )\cdot \;\theta_{i} \end{array}$$

In Model 3, we used subject-specific mixing weights β_i_ that combine a single inflection point 𝜃 with task-specific parameter α_j_

$$\begin{array}{c} \mathrm{logit}(k_{ij})\sim \;\beta_{i}\cdot \;\alpha_{j}+(1-\beta_{i})\cdot \;\theta \end{array}$$

**FIGURE 4:**
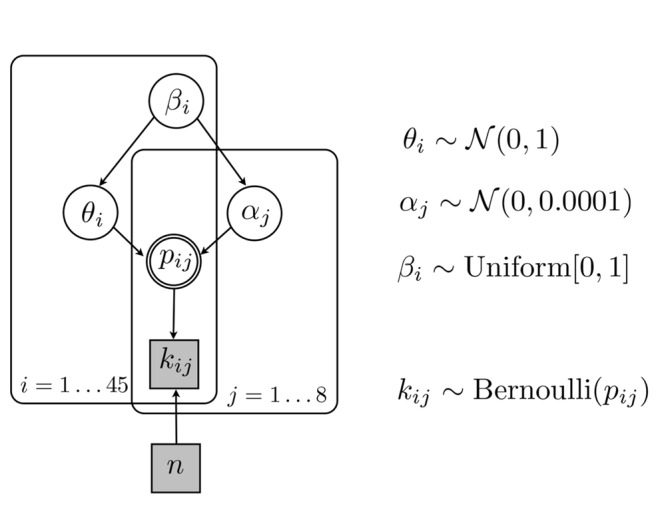
**Graphical illustration of the Bayesian Logistic Mixed Model with subject-specific mixing and inflection points (Model 4)**. Parameters of interest are task-specific α_j_, but also subject-specific β_i_ and 𝜃_i_. Discrete binary observed parameters are shown in gray squares whereas continuous estimated parameters are shown in white circles and the double circle denotes probabilities that can be determined. Plate notation with indices *i* and *j* groups the subject- and task-specific parameters, respectively.

Model 4 is illustrated in **Figure 4** and postulates a mixed model with subject-specific weights β_i_ and inflection points 𝜃_i_

$$\begin{array}{c} \mathrm{logit}(k_{ij})\sim \;\beta_{i}\cdot \;\alpha_{j}+(1-\beta_{i})\cdot \;\theta_{i} \end{array}$$

All variants of the Bayesian logistic model assumed a non-informative prior for task-specific parameters α_j_, a uniform prior for (subject-specific) mixing parameters β_(i)_, and a weakly informative prior for (subject-specific) inflection point 𝜃_(i)_. The latter parameter was centered on 0 in log odds (equivalent to probability 0.5) because in each test round the participant was asked to choose between two suits (on target or off target).

In a first step we established which model had the least predictive error in terms of DIC. The DIC is a hierarchical modeling generalization of the AIC and Bayesian Information Criterion (BIC). As most information criteria the DIC lacks a clear theoretical foundation (Plummer, 2008) but has proven useful in Bayesian model selection problems where the posterior distributions of the models are obtained by MCMC simulation (Lunn et al., 2000; Spiegelhalter et al., 2014).

Similar to AIC and BIC, DIC is an asymptotic approximation as the sample size becomes large. However, DIC is only valid when the posterior distribution is approximately multivariate normal (Gelman et al., 2013). DIC values should not be interpreted in absolute terms but a lower DIC value in a model comparison indicates a more parsimonious model fit. Depending on the specific application a difference in DIC of more than 5 is considered meaningful.

Not surprisingly the model with the lowest deviance (^D) was the logistic mixed Model 4 with subject-specific mixing weights and subject-specific inflection points and the model with the highest deviance was Model 1 with a single mixing weight and inflection point. Model 1 allows no subject-specific variability whereas Model 4 appears to overfit the data. The most promising models are Models 2 and 3 but the most parsimonious model with the lowest DIC value was Model 3 (DIC_Model__4_–DIC_Model__3_ = 391.6–361.2 = 30.4). This model combines task-specific parameters with a constant inflection point using subject-specific mixing parameters. The model selection results are summarized in **Table 1**.

### MCMC Simulations

Parameters 𝜃_i_ were drawn from a weakly informative prior, a normal distribution on log odds centered on 0 and precision 1. The normal distribution is a plausible prior for the latent variables but it is not a conjugate for the likelihoods in a logistic model. This makes it difficult to derive posterior distributions without MCMC sampling.

The task-specific log odds α_j_ were drawn from a non-informative prior, a normal distribution of log odds centered on 0 and a precision of 0.0001, whereas the mixing parameters β_i_ ∈ [0,1] were sampled form a uniform distribution between 0 and 1. This constrains transformations of α_j_ to inverse-S or concave shapes.

In a hierarchical model extension we also introduced a hyper-parameter for the standard deviation *σ* (gamma-distributed parameter *τ* for precision) of parameter 𝜃_i_ ∼𝒩(0,τ) of the task-specific prior distribution (Lee and Wagenmakers, 2013). The hyper-parameter reduced the credible intervals of parameter estimates but increased the predictive error of all models and was therefore not considered further.

Using the binary gaze directions and choice predictions from *N* = 45 participants in four tasks, we ran MCMC simulations for each Bayesian logistic model using the Metropolis–Hastings algorithm as implemented in WinBUGS 1.4 (Lunn et al., 2000; Lee and Wagenmakers, 2013) with an interface to R (R2WinBUGS) to obtain posteriors and credible intervals for the parameters of interest as well as missing values. We defined three chains with 10,000 iterations each. The length of burn-in was set to 1,000 and thinning to 1. We specified different initial values for each chain (see Appendix A.2 for BUGS model and Appendix A.3 for illustration of output).

Comparing within and between variability of the chains and traces in the MCMC simulations indicated convergence on a single posterior distribution (^R = 1.0) for all model parameters and models including Model 4. We also checked auto-correlations and found no notable correlation across lags. With convergence assured, we established posterior distributions and credible intervals for the parameters of interest.

### Two Systems ToM

The Bayesian Logistic Mixed Model 3 with subject-specific mixing weights and single inflection point gave the best model fit in terms of DIC. Model 3 also outperformed all tested logistic regression models with mixed effects. We therefore used this Bayesian model to test the hypothesis whether implicit eye-gaze and explicit choice-prediction are better represented by a single or two ToM systems. More specifically, we compared a two-system ToM model with eight independent task-specific parameters (four for eye gaze and four for choice prediction) against a single-system ToM model with only four task-specific parameters – one parameter per task for eye gaze direction and choice prediction. The DIC clearly indicates that a two-systems ToM with independent sets of latent parameters captured the data better than a single-system ToM (DIC_1Sys_ = 410.2–DIC_2Sys_ = 361.3 = 48.9).

### Registrations and Alternative Hypothesis

We also tested the Registration vs. Alternative hypothesis using eye gaze as a proxy for implicit belief tracking by the fast and efficient system. For the Registrations model we equated the task parameters for TB, FB, and Ignorance (TB = FB = IG) for eye gaze but assumed a separate parameter for CL because this task relates to first-order rather than second-order perspective taking. For the Alternative Model we equated the task parameters for FB and Ignorance (FB = IG) for eye gaze. A comparison between Alternative and Registration model with differently constrained task-specific parameters and subject-specific mixing weights favored the Alternative over the Registrations hypothesis (DIC_Reg_–DIC_Alt_ = 369.8–362.6 = 7.2).

In the following we contrast the results of Model 2 with the results of Model 3 because they give different accounts of probability weighting in log-odds and suggest different subject-specific characteristics.

### Bayesian Logistic Mixed Model 2

**FIGURE 5:**
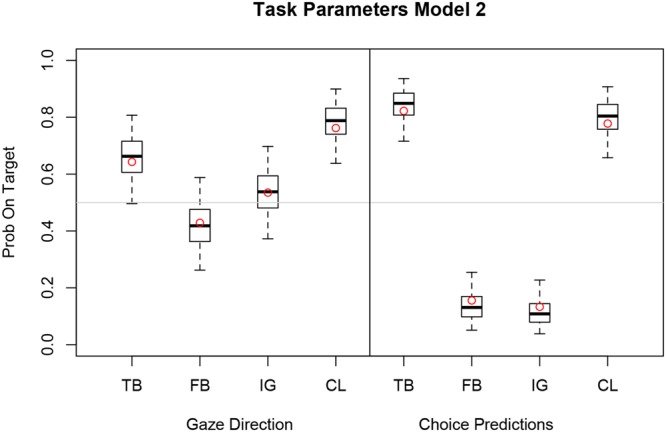
**Boxplot of estimated task parameters for eye gaze and choice prediction with median (horizontal bar), 50% (box) and 95% (whisker) credible intervals.** Task parameters (TB, FB, IG, CL) of Model 2 with subject-specific inflection point 𝜃_i_ and mixing weight β are expressed as probabilities on target.

The box plot in **Figure 5** shows the transformed log odd estimates

$$\begin{array}{c} \mathrm{Prob}(\mathrm{task}\;j)=\frac{\exp(\alpha_{j})}{\exp(\alpha_{j})+1} \end{array}$$

of the task parameters for gaze direction and choice prediction.

Estimates are expressed as probability on target for TB, FB, Ignorance (IG), and Control (CL) of Model 2. Observed relative frequencies on target for each measure and task are superimposed as red dots.

For eye gaze the TB, FB, and IG estimates are close to chance level (0.5) but follow a similar pattern as the estimates for choice predictions. Note that the means of the task-specific parameters correspond closely to the observed relative frequencies (red dots).

**FIGURE 6:**
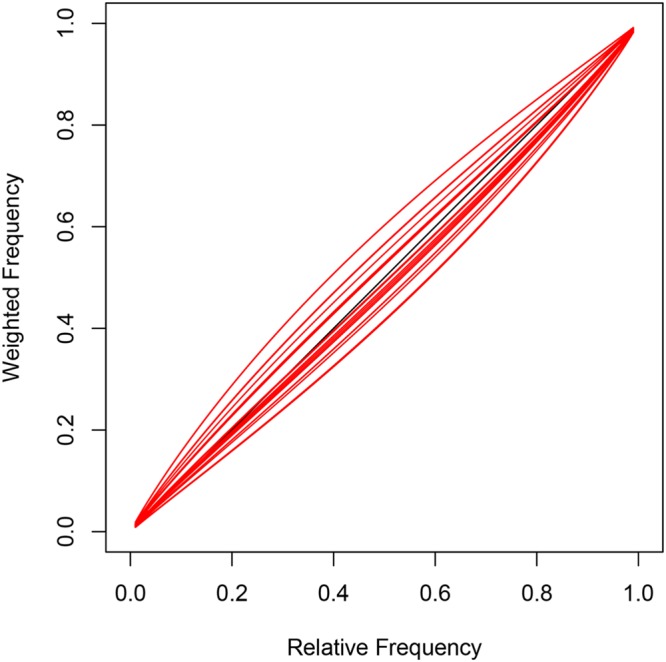
**Task-specific parameters and subject-specific inflection points 𝜃_i_ in Model 2 lead to slightly distorted individual probability functions (red curves)**.

Model 2 has task-specific parameters that do not deviate from the observed relative frequencies for each task. This is a consequence of the individual weighting functions (red curves) that are only slightly distorted from the diagonal suggesting good discrimination between task-specific probabilities in all participants (see **Figure 6**). In summary, the task-specific parameters indicate clear first-order and second-order perspective taking for choice predictions. Initial gaze direction however, reflects first-order perspective taking (CL) but not necessarily second-order perspective taking (TB, FB, and IG).

### Bayesian Logistic Mixed Model 3

**FIGURE 7:**
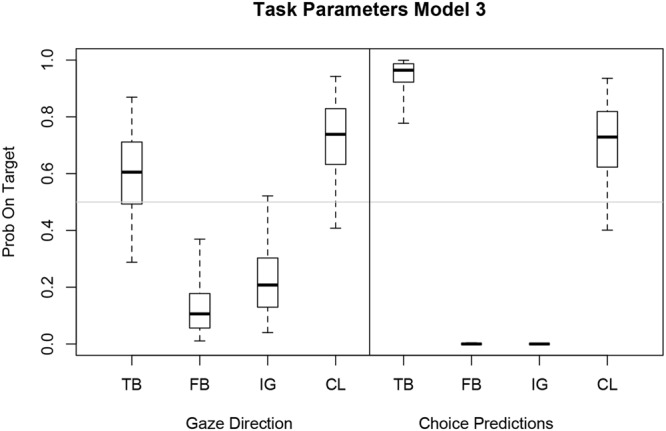
**Boxplot of estimated task parameters for eye gaze and choice prediction with median (horizontal bar), 50% (box) and 95% (whisker) credible intervals**. Task-specific parameters of Model 3 expressed as probabilities on target for gaze direction and choice prediction. Model 3 has a fixed inflection point 𝜃 and subjective-specific mixing β_i_.

**Figure 7** shows task-specific parameter estimates of Model 3 for gaze direction and choice prediction transformed from log odds to probabilities on target. The credible intervals for the explicit choice predictions are clearly different for TB compared to FB and IG task. The negative log odds for FB and IG give probabilities on target that are almost at 0 whereas the positive log odds for TB result in a probability of 0.97 (right panel of **Figure 7**). A similar but less pronounced pattern emerges for gaze direction (left panel of **Figure 7**). The probability estimate on target for TB equals 0.61 whereas FB and IG have probabilities of 0.10 and 0.20, respectively. The CL task has a probability on target of 0.75 for eye gaze and 0.74 for choice prediction.

Interestingly, the CL estimates are almost identical for gaze direction and choice prediction and also correspond to the estimated inflection point *𝜃* = 0.75. Comparing eye gaze direction with choice prediction, the TB, FB, and IG estimates for choice prediction are less extreme and have larger credible intervals but follow a similar general pattern as the task-specific estimates for choice prediction.

**FIGURE 8:**
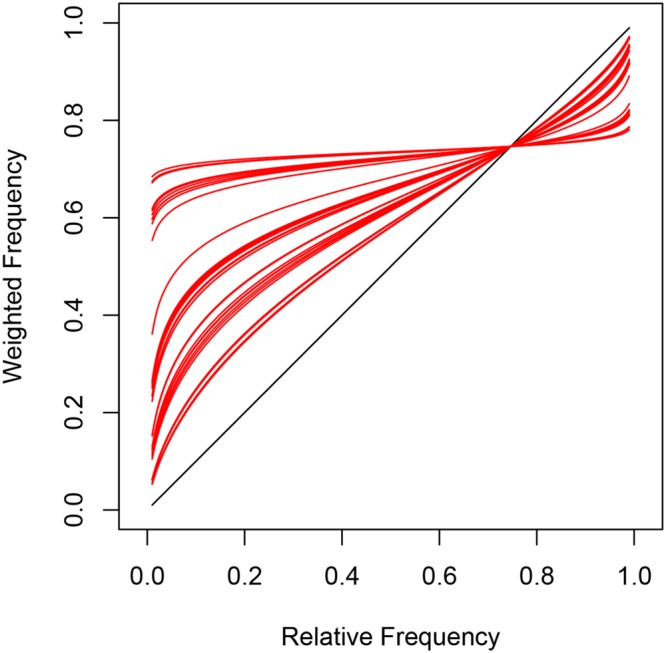
**Subject-specific mixing of task-specific parameters and inflection point in Model 3 leads to inverse S-shaped functions (red) that are typical for subjective probabilities in decision-making under risk and uncertainty**.

The weighting functions in **Figure 8** are similar to subjective probability curves reported by Kahneman and Tversky (1979), generalized by Prelec (1998) and Luce (2000), and also investigated by Zhang and Maloney (2012). Note that inverse S-shaped functions are typical for subjective probability in decision making under risk and uncertainty. The inflection point at 0.75 suggests an overall tendency toward the target for both implicit and explicit measures. Task-specific probabilities below *𝜃* = 0.75 were overestimated whereas probabilities above 0.75 were underestimated but with a different probability curve for each subject.

The gradual increase in steepness of the inverted S-shaped probability functions (**Figure 8**) suggests different degrees of discrimination between task-specific beliefs across participants. Some participants have probability functions that are almost flat or horizontal indicating very poor discrimination between task probabilities. Most participants, however, show a steeper increase in their functions and therefore better understanding of task-specific differences. All participants shared the characteristic of overestimating frequencies below the inflection point at 0.75 and underestimating frequencies above the inflection point. Interestingly, the inflection point matches the task-specific CL parameter α_4_ = 0.75 for eye gaze as well as α_8_ = 0.74 for choice prediction. Both task parameters describe the tendency for looking at or choosing the target according to first-order perspective taking. The CL estimates and *𝜃* suggest that the default for looking at and choosing a target card was higher than chance level and similar for implicit and explicit measures across all observers.

## Discussion

The specific aim of the present study was to test how well adults can track belief states in others. Two main hypotheses were put forward: (a) Adults use a fast and efficient ToM system for implicit belief tracking that may be similar to infants and (b) this system is different from explicit belief tracking.

Both hypotheses imply a fast and efficient ToM system (Apperly and Butterfill, 2009) that can explain at least first-order perspective taking and possibly some second-order perspective taking (Hamlin et al., 2013).

If the fast and efficient system relies on so-called registrations, initial eye gazes on and off target should be the same in tasks that require tracking of beliefs about mental states, no matter if these beliefs are true or false (Registrations hypothesis). If, however, the fast and efficient system relies on different mechanisms that allow tracking of mental states then participants’ gaze directions may even discriminate between TB and FB tasks (Alternative hypothesis).

Logistic regression models with mixed effects performed well but a Bayesian logistic mixed model with subject-specific mixing of latent task-specific parameters with a constant inflection point provided a better account of the data. Bayesian Logistic Mixed Model 3 suggests two systems and favored the Alternative over the Registrations model hypothesis.

According to Model 3 the latent task parameters for eye gaze were neither at chance nor did they match explicit choice predictions. Importantly, they also did not reflect a simple egocentric bias (TB = FB = IG = CL). Implicit eye gaze as well as explicit choice predictions were anchored at 0.75 in the CL task indicating first-order perspective taking whereas task-specific probabilities on target as low as 0.1 and 0.2 in the FB and IG task respectively suggest second-order perspective taking.

Probabilities on target for choice predictions but also for eye gaze discriminated between TB and FB tasks suggesting second-order perspective taking in both measures. Not surprisingly, explicit choice prediction showed a clear appreciation of different belief states in others whereas initial gaze direction reflected reduced second-order perspective taking.

Although, the Ignorance (IG) match was constructed in such a way that Player 2 would remain ignorant about the target suit, participants’ choice predictions in the IG task were not at chance level. Similar to the FB task participants predicted that Player 2 would choose the non-target rather than the target suit. Player 1 did not try to deceive Player 2 as in the FB match but chose the same non-target suit twice. Therefore participants may have assumed that Player 2 engaged in a “probability matching” strategy. Probability matching is a simple heuristic whereby Player 2 simply picks the suit that has been selected most by the opponent (Player 1). Participants may follow this strategy when predicting that Player 2 selects the non-target suit in the IG task.

If Player 1 had chosen each suit only once in the four rounds of the IG match then Player 2 would have been truly ignorant about the target suit. Such a match would have been a better scenario to suggest ignorance in Player 2.

Future studies may investigate how adults track TB/FB over successive rounds. If observers accumulate evidence in favor of TB or FB across rounds then this may be reflected in their initial eye gaze as a measure of implicit belief tracking. It would be interesting to record initial eye gaze over successive rounds to monitor whether observers’ updated prior odds are reflected in their initial eye gaze. This updating process may serve as an explanation for the similarities between implicit and explicit belief tracking in the present card game. Updated prior information may not only affect explicit choice predictions but also initial eye gaze. Increased variability or noise in eye gaze may have obscured the fact that adult participants rely on explicit belief tracking when implicitly tracking the beliefs of others.

Despite these limitations, the present experimental paradigm and theoretical approach appears promising to investigate implicit and explicit tracking of beliefs in others. Our results on a fast and efficient system are not conclusive but they seem to be in line with the findings reported by Hamlin et al. (2013). In their study, preverbal infants judged agents by the agents’ knowledge about the goal of another character. This supports the view that adults’ implicit belief tracking could be guided by an implicit system similar to infants’ social judgments. However, Hamlin et al. (2013) did not include a false-belief condition in their study.

If infants’ and adults’ implicit performances correspond then predicted signature limits of the fast and efficient system as proposed by Apperly and Butterfill (2009) need to be reconsidered. Converging results from infants’ and adults’ implicit performance that also resemble adults’ explicit performance supports the view that the fast and efficient implicit system is more capable of belief tracking than previously suggested. Alternatively, it is also possible that explicit belief tracking influenced implicit belief tracking in the present card game.

A critical question is whether there is a scenario that can lead to opposite implicit and explicit belief tracking. The present card game provides a versatile tool to create various perspective-taking and belief-tracking situations. Different scenarios in this card game may be developed into a diagnostic tool for evaluating ToM abilities in typical and atypical populations. Possible applications include research on individual differences in autism spectrum disorder (ASD). Impaired ToM abilities are an important issue in ASD, but there is still controversy about how far these problems are mediated by motivational, attentional, and other factors (e.g., Peterson et al., 2013). One advantage of the present card game is that it does not involve social cues that may give typical participants an advantage over participants with ASD.

## Conclusion

The card game in combination with Bayesian logistic mixed models is a powerful tool to investigate implicit and explicit belief tracking abilities in adults. Bayesian logistic mixed models offer subject- and task-specific parameter estimates with credible intervals even when the sample is small and data are missing. These models are more flexible than logistic regression analyses and can reveal characteristics in the data that would be missed otherwise. However, Bayesian models need to be established carefully and need to undergo thorough checks to make sure that MCMC sampling results are not an artifact of the model constraints.

At first glance the results from adult participants seem to add evidence to a growing body of research that supports implicit belief tracking as part of a two-system ToM. On closer inspection however, after employing a Bayesian logistic model, initial gaze direction as a measure of implicit belief tracking appears to reflect explicit choice prediction. Further research needs to examine whether adult participants are genuinely able to implicitly track TB/FB or whether implicit belief tracking reflects second-order perspective taking as a consequence of a belief updating process across rounds. Inferring what others are thinking and intending to do remains one of the most fascinating but also puzzling abilities of a socially intelligent mind. At least in scientific terms we are beginning to develop interactive paradigms and quantitative tools that help to unravel the complex processing associated with belief tracking as part of ToM.

## Author Contributions

All authors listed, have made substantial, direct and intellectual contribution to the work, and approved it for publication.

## Conflict of Interest Statement

The authors declare that the research was conducted in the absence of any commercial or financial relationships that could be construed as a potential conflict of interest.
