# Supplementary material for: Logistic Mixed Models to Investigate Implicit and Explicit Belief Tracking
Source: Front Psychol. 2016 Nov 2;7:1681. doi: 10.3389/fpsyg.2016.01681 (PMC5090957; doi:10.3389/fpsyg.2016.01681)
Supplement: Supplementary file 1 [file Data_Sheet_1.docx]

Appendix

Appendix A.1 Results of Logistic regression with mixed effects (lmer pars)

Generalized linear mixed model fit by maximum likelihood (Laplace Approximation) [glmerMod]

Family: binomial ( logit )

Formula: dv ~ (TB.c + FB.c + IG.c) * meas.c + (0 + meas.c | subj)

Note: dv = binary dependent variable (1= on target, 0 = off target). Dummy coding for contrasts between centralized task (TB, FB, IG) and CL as baseline. The model was fitted by maximum likelihood (Laplace Approximation) in R 3.1.1 using the lme4 package (version 1.1-7) with glmer (family='binomial', control= glmerControl(optimizer='bobyqa').

AIC BIC logLik deviance df.resid

399.7 434.3 -190.8 381.7 340

Random effects:

Groups Name Variance Std.Dev.

subj meas.c 0 0

Number of obs: 349, groups: subj, 45

Fixed effects:

Estimate Std. Error z value Pr(>|z|)

(Intercept) 0.1030 0.1292 0.797 0.42532

TB.c 0.1483 0.3590 0.413 0.67949

FB.c -2.1976 0.3627 -6.059 1.37e-09 ***

IG.c -2.0740 0.3693 -5.616 1.96e-08 ***

meas.c 0.5956 0.2583 2.305 0.02114 *

TB.c x meas.c 0.8541 0.7179 1.190 0.23420

FB.c x meas.c 1.4936 0.7253 2.059 0.03947 *

IG.c x meas.c 2.1012 0.7386 2.845 0.00444 **

---

Signif. codes: 0 ‘***’ 0.001 ‘**’ 0.01 ‘*’ 0.05 ‘.’ 0.1 ‘ ’ 1

Correlation of Fixed Effects:

(Intr) TB.c FB.c IG.c meas.c TB.c:. FB.c:. IG.c:.

TB.c 0.006

FB.c 0.009 -0.499

IG.c 0.034 -0.490 0.485

meas.c -0.203 0.069 -0.100 -0.133

TB.c x meas.c 0.069 -0.089 -0.005 -0.005 0.006

FB.c x meas.c -0.100 -0.005 -0.132 0.005 0.009 -0.499

IG.c x meas.c -0.133 -0.005 0.005 -0.176 0.034 -0.490 0.485

Appendix A.2 Bayesian Logistic Mixed Model specification in BUGS

model{

for (i in 1 : np) {

for (j in 1 : nq) {

k[i, j] ~ dbern(p[i,ind[j]]) # ind[]=index for grouped tasks

}

}

for(i in 1:np){ # index for subjects

for (z in 1:nz){ # index for tasks

logit(p[i, z]) <- beta[i] * alpha[z] + (1-beta[i]) * theta

}

beta[i] ~ dunif(0,1) # uniform prior for subject-specific intercept

}

# prior for grouped task-specific parameters

for (z in 1:nz){

alpha[z] ~ dnorm(0.0, 0.0001)

}

theta ~ dnorm(0,1) # intercept centered on 0 log odds = 0.5

# 11 missing data in binary data matrix k[i, j]

NA.array[1] <- k[9,2]

NA.array[2] <- k[10,1]

NA.array[3] <- k[10,4]

NA.array[4] <- k[11,2]

NA.array[5] <- k[11,3]

NA.array[6] <- k[16,1]

NA.array[7] <- k[20,3]

NA.array[8] <- k[27,4]

NA.array[9] <- k[40,1]

NA.array[10] <- k[40,2]

NA.array[11] <- k[40,4]

}

Appendix A.3: Summary and illustration of Bayesian Logistic Mixed Model 3 estimates
